# Evaluation of Titanium Serum Levels in Patients After Spine Instrumentation: Comparison Between Posterolateral and 360º Spinal Fusion Surgery

**DOI:** 10.7759/cureus.5451

**Published:** 2019-08-21

**Authors:** Ignacio Fernández Bances, José Paz Aparicio, Marco Antonio Alvarez Vega

**Affiliations:** 1 Spine Unit, Orthopedic Department, Complejo Asistencial Universitario De León, León, ESP; 2 Spine Unit, Orthopedic Surgery and Traumatology Department, University Central Hospital of Asturias, Oviedo, ESP; 3 Neurosurgery Department, University Central Hospital of Asturias-Huca, Oviedo, ESP

**Keywords:** serum titanium levels, spine fusion, interbody devices, metal ion release, isotope dilution analysis

## Abstract

Introduction

The use of orthopedic implants is a cause for concern for the release of its integrating metals and the systemic complications that may occur. Instrumented spine arthrodesis is the recommended treatment for many spine diseases. Different segmental fixation devices, commonly made of titanium and its alloys, are used in these surgeries. The use of this metal for spinal fusion has introduced the possibility of generating microscopic metal particles that are present in the tissues of the surrounding implants (peri-implant environment). In fact, metal debris has been found in the paraspinal soft tissues of patients with posterior lumbar instrumentation and showed to be particularly high in patients undergoing revision procedures of pseudoarthrosis. In addition, part of the metals might also dissolve (either from the released particles or directly from the implant surface) and circulate in the body fluids, accumulating (eventually) in remote organs.

Material and methods

A prospective study was designed with patients who were to be operated by the pathology of the lumbar spine to perform a vertebral arthrodesis composed of a titanium alloy (n=32). Two subgroups were differentiated according to the type of surgery performed: a) Posterolateral arthrodesis (N=5); b) Circumferential arthrodesis intervertebral implant of polyester-ether ketone (PEEK) (N=8) or titanium (N=19). The blood sample was taken before surgery and one year later. The samples were analyzed by mass spectrophotometry with a double focus inductive coupling plasma source (DF-ICP-MS).

Results

Blood titanium levels prior to surgery were similar to those in other publications (0.7449 micrograms per liter^-1 ^(µgL^-1^), Standard Deviation (SD)=0.562). The average titanium concentration levels found after surgery was 2.5406 µgL^-1 ^(SD=3,69), near 3.5-fold increase. After surgery, there was a significant mean increase in serum titanium levels of 1.7957 µgL^-1 ^(SD=3.5765, Range=-0.57 µgL^-1^; 14.60 µgL^-1^). There is a statistically significant increment (p=0.00049) of the titanium concentration in the serum of the patients after surgery. If we analyze the patients in three groups according to the type of implants used (posterolateral, circumferential with PEEK, and circumferential with titanium), there are no differences between those who did not have an intersomatic device implanted and those in which PEEK implants were implanted, but with those in which it was titanium it was p=0.006 and p=0.018, respectively.

Conclusions

Patients undergoing vertebral instrumentation experience a significant increase in serum titanium levels compared to before surgery levels. The use of an intersomatic device did not show differences in titanium release with not using it when it was PEEK. There are significant differences between patients without intersomatic implants or those who had a PEEK implant with those in whom it was titanium, with a significant increase in blood titanium levels.

## Introduction

The metallic implants placed in human bodies can exhibit electrochemical erosion that yields in the liberation of metallic products. Such implants-derived metal wear products can be present in the form of metal ions and/or small nanoparticles with still unknown effects on human health.

Instrumented spinal arthrodesis is the recommended treatment for progressive spine deformity and degenerative disc disease. For these kinds of surgeries, different segmental fixation devices, including hooks, screws, wires, etc., commonly made of titanium and its alloys, are used [1]. The use of this metal for spinal fusion has introduced the possibility of generating microscopic metal particles, which are present in the tissues of the surrounding implants (peri-implant environment) [2]. In fact, metal debris has been found in the paraspinal soft tissues of patients with posterior lumbar instrumentation and showed to be particularly high in patients undergoing the revision procedures of pseudoarthrosis [3]. These particles (nanometer size) activate macrophages that increase bone absorption and inflammatory reactions [4]. In addition, part of the metals might also dissolve (either from the released particles or directly from the implant surface) and circulate in the body fluids, accumulating (eventually) in remote organs [5]. But metallic prostheses and orthopedic implants are known to provide also nano-debris that is generated at the bone-biomaterial interface into the microenvironment [6]. Studies have shown that metallic debris can induce bone loss, loosen implants, and sometimes cause the clinical failure of bone prostheses [7]. In addition, there is growing evidence that debris can directly affect the cells of the osteoblast lineage [8]. The possible harmful effects of titanium oxide nanoparticles on their local environment and on the health of the patients are still uncertain, therefore, the assessment of their risk is necessary, both in vivo and in vitro. In vitro examination using cultured cells is frequently used as a powerful tool for screening hazardous materials, and it is essential for understanding the mechanisms of the biological influences induced by nanoparticles [9]. Therefore, the adverse effects of titanium peri-implant cells, especially bone cells, should be determined. Additionally to the production of nanoparticles, the degradation products of all orthopedic implants also generate metal ions (either from direct electrochemical corrosion of the implant surface or through the dissolution from metallic nanoparticles as intermediates) [10]. Together with the production of nanoparticles, there is continuing concern regarding the release of chemically active metal ions, which bind to proteins and remain in solution from which they can then disseminate into the surrounding tissues, bloodstream, and remote organs [11-12]. Particulate metallic wear debris presents an enormous surface area for electrochemical dissolution, which is a major factor contributing to the observed systemic elevations in the metals of patients with titanium implants [13].

In the present study, our objective is to determine the variation in serum titanium levels in patients undergoing instrumented spinal arthrodesis surgery. Also, we will evaluate the different types of implants in order to establish a possible correlation between the liberation rate and the micromotion of the particular metallic device. For this analysis, is important to use highly sensitive analytical techniques that permit to detect ultratrace concentration levels of titanium and its quantification with high precision and accuracy. In this case, double focussing elemental mass spectrometry (DF-ICP-MS) has provided satisfactory results in previous studies and will be applied also here using isotope dilution analysis for quantification purposes [14-15].

## Materials and methods

A prospective study was designed, which included, in the case group, patients older than 18 years old belonging to the Health Area of the Central University Hospital of Asturias (Oviedo-Spain), with a population close to 350,000 people, who were to be operated by the pathology of the lumbar spine to perform a vertebral arthrodesis with instrumentation composed of a titanium alloy. All patients underwent surgery between August 2011 and October 2012 by the same team of two senior spine surgeons. Two subgroups were differentiated according to the type of surgery performed:

a) Posterolateral arthrodesis (N=5)

b) Circumferential arthrodesis intervertebral implant of polyester ether ketone (PEEK) (N=8) or titanium (N=19)

The same patients in the case group were included in the control group, prior to surgery determining basal blood titanium levels. The inclusion criteria were (Table 1):

**Table 1:** Inclusion criteria in the study

INCLUSION CRITERIA
Healthy patient over 18-years-old belonging to the Health Area of the Central University Hospital of Asturias (Oviedo-Spain), without relevant medical or surgical history.
Suffer from a spinal pathology with indication of spinal fusion surgery.
Not be a carrier of any type of metallic implant, be it aesthetic or therapeutic.
Not having any skin tattoo or piercings.
Not suffer any type of mental or physical illness that may interfere with the recovery process of his pathology.
Give consent for inclusion in the study by signing the approval document of the Medical Ethics Committee of our hospital for the collection of a blood sample and inclusion in the study.

Samples

**Figure 1:**
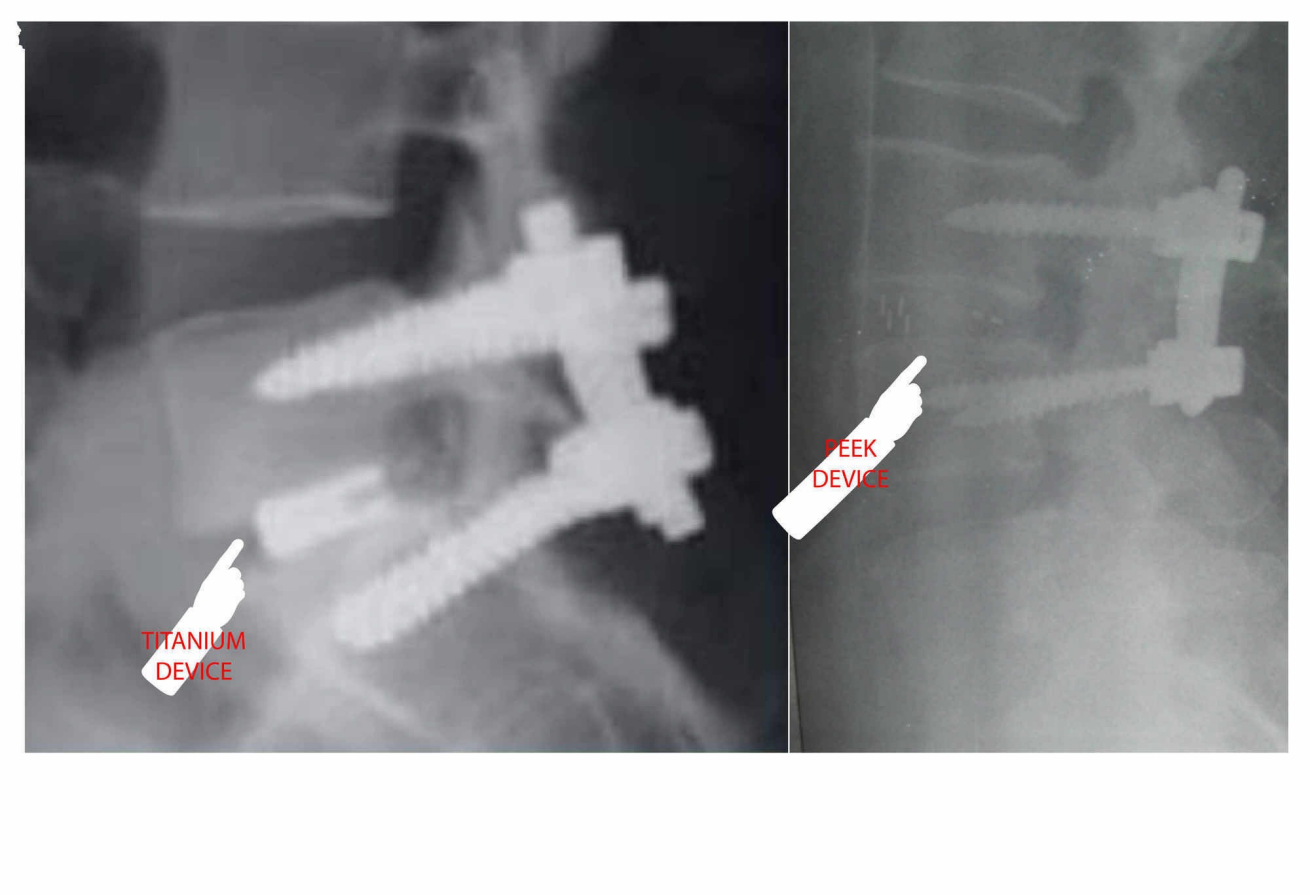
Radiography picture from a circumferential spine fusion with titanium (picture on the left) and PEEK (picture on the right) PEEK: polyester ether ketone

Serum from patients (32 subjects) was collected before and after (one year) posterior spinal arthrodesis. Two types of spinal fusion implants have been evaluated and compared: posterolateral spinal fusion in which the bone graft is placed between the transverse processes and the facet joint in the back of the spine. These vertebrae are then fixed in place with titanium screws through the pedicles of each vertebra attaching to a metal rod (also made of titanium) on each side of the vertebrae. On the other hand, interbody spinal fusion places the bone graft between the vertebra in the area usually occupied by the intervertebral disc and between the transverse processes. In preparation for the spinal fusion, the disc is removed entirely and a device is then placed between the vertebrae to maintain spine alignment and recover the height of the disc. Such an intervertebral device is made from either PEEK or titanium. The fusion then occurs between the endplates of the vertebrae. Using both types of fusion is known as 360-degree fusion (Figure 1). The analyzed samples in this work corresponded to three different groups of patients undergoing: posterolateral fusion (five patients) and 360-degree fusion (25 patients) out of which eight patients carried a peek intervertebral device and 19 patients carried a titanium intervertebral device. PEEK intervertebral devices used are named Capstone^TM^ and manufactured by Medtronic (Memphis, TN, USA). Titanium intervertebral devices are named Prospace^TM^ and they are made of titanium, aluminum and vanadium alloy (Ti6Al4V) and manufactured by Braun-Aesculap (Tuttlingen, Germany). The screws and rods used belong to the CD-Horizon​​​​​^TM^ Legacy^TM^ system manufactured by Medtronic (Memphis, TN, USA) and S4^TM ^Spinal System manufactured by Braun-Aesculap (Tuttlingen, Germany), both made of Ti6Al4V alloy.

The samples were collected in 5 milliliters (mL) vacutainer tubes (Greiner Bio-One^TM^, Madrid, Spain) for trace elements. The precautions taken to avoid any metal contamination during blood extraction have been described before and those procedures were followed here [14]. Briefly, blood samples were withdrawn from a forearm vein using a stainless-steel needle surrounded by an inert plastic cannula. Blood was drawn into standard plastic syringes. The first 5 mL of blood extracted from each patient were used to rinse the system and were then discarded. The second 5 mL was transferred to blood-collection tubes conditioned with lithium heparin (Vacuette^TM^) and used to conduct the analysis. The samples were transferred to an accredited laboratory at the Analytical Chemistry Department of the University of Oviedo. Immediately after collection, the blood samples were separated into serum and red blood cells by centrifugation at 3000 g for 15 minutes. Serum samples obtained in this way were stored at -20° C until analysis.

Serum samples preparation

Serum samples (n=32) used to determine the total concentration of titanium were prepared following the method used in previous studies [16]. The serum was just diluted before analysis as follows: 600 microliters (μL) of serum was mixed with 30 μL of the spike solution: (11.5 micrograms per liter^-1 ^(µgL^-1^) titanium isotopically enriched on the isotope titanium-47 (^47^Ti) and diluted to a final volume of 3 mL with ultrapure water. No spiking of the samples was conducted at any point of this particular study.

A double focusing-sector field elemental mass spectrometry instrument (DF-ICP-MS), ELEMENT 2 (Thermo Fischer Scientific, Inc., Waltham, MA), was used for the total titanium determination using medium resolving power (m/Dm=3000). The instrument was equipped with a Meinhard concentric glass nebulizer and with nickel sampler and skimmer cones.

The detection limit of the applied methodology (0.05 µgL^-1^) together with the suitability of the proposed quantification strategy has been established in a previous publication by means of a certified reference material [15].

Statistical analysis

Categorical variables are presented as frequencies and percentages, and continuous variables as means and standard deviations or medians and ranges. A paired t-test was used to evaluate the difference in titanium levels preoperatively and one year after surgery. The independent samples Kruskal-Wallis test was used to compare between groups. All reported p-values are two-sided, with a significance of 0.05. All analyses were performed with the use of IBM^TM ^SPSS^TM ^Statistics 22.0 Software (IBM Corp, Armonk, NY, USA).

Study registration and informed consent

This study was checked and approved by the Regional Clinical Research Ethics Committee of Principado de Asturias located at University Central Hospital of Asturias (Oviedo-Spain). The patients participated in this study voluntarily, and they all were informed about the objective of the work. Written informed consent was obtained from all patients to take part in this study and collect the samples, and another informed consent was signed for surgery. Samples were collected in accordance with protocols approved by the relevant institutional review boards and with the Helsinki Declaration of 1975, as revised in 2000.

## Results

Initially, 44 patients who met all the previously established inclusion criteria, obtaining the first pre-surgical sample of all of them. However, 12 patients did not show up to the blood draw one year after surgery, so they were excluded from the study because they did not complete the study protocol. Finally, 32 patients were included for the analysis of the data, with a mean age of 55.31 years (Standard Deviation (SD)=9.670) at the time of surgery.

Twelve were male (37.5%) and 20 female (62.5%). In relation to the types of surgery, five patients (15.6%) underwent posterolateral vertebral arthrodesis and 25 patients (84.4%) circumferential vertebral arthrodesis, of which eight PEEK devices (25%) were implanted and 19 titanium devices (59.4%) were used. In all cases, titanium rods and screws were applied. Demographic data are shown in Table 2.

**Table 2:** Demographic data and serum titanium levels before surgery and after surgery µg L^-1^: micrograms per liter^-1^; L4-L5: Fourth lumbar vertebra-fifth lumbar vertebra; L5-S1: Fifth lumbar vertebra-first sacral vertebra; PEEK: polyester ether ketone; T12-L1: Twelfth thoracic vertebra-first lumbar vertebra

NUMBER OF PATIENT	SEX	AGE AT TIME OF SURGERY	DIAGNOSIS	INTERBODY DEVICE	PRESURGICAL SERUM TITANIUM LEVELS (µg L^-1^)	POSTSURGICAL SERUM TITANIUM LEVELS (µg L^-1^)
Patient 1	Female	69	Lumbar Canal Stenosis L4-L5	Titanium	3,2 ± 0,4	9,74 ± 0,18
Patient 2	Male	55	Lumbar Canal Stenosis L4-L5	Titanium	0,37 ± 0,05	14,81 ± 0,21
Patient 3	Female	57	Degenerative Disc Disease L5-S1	No	0,8 ± 0,1	1,365 ± 0,59
Patient 4	Female	73	Lumbar Canal Stenosis L4-L5	Titanium	0,65 ± 0,08	1,7 ± 0,1
Patient 5	Female	66	Lumbar Canal Stenosis L4-L5	Titanium	1,78 ± 0,8	1,7 ± 0,4
Patient 6	Female	58	Lumbar Spondylolisthesis L4-L5	Titanium	0,7 ± 0,2	2,481 ± 0,024
Patient 7	Male	45	Lumbar Spondylolisthesis L4-L5	Titanium	0,51 ± 0,06	1,600 ± 0,006
Patient 8	Male	50	Degenerative Disc Disease L5-S1	Titanium	0,9 ± 0,2	15,50 ± 0,59
Patient 9	Male	45	Degenerative Disc Disease L5-S1	Titanium	0,93 ± 0,07	1,3 ± 0,4
Patient 10	Female	67	Degenerative Disc Disease L5-S1	Titanium	0,53 ± 0,06	1,81 ± 0,13
Patient 11	Female	42	Lumbar Canal Stenosis L4-L5	Titanium	0,23 ± 0,02	1,263 ± 0,002
Patient 12	Female	65	Degenerative Disc Disease L5-S1	Titanium	0,24 ± 0,08	2,24 ± 0,04
Patient 13	Female	52	Degenerative Disc Disease L4-L5	PEEK	0,24 ± 0,07	0,85 ± 0,09
Patient 14	Male	50	Degenerative Disc Disease L5-S1	Titanium	0,93 ± 0,02	2,3 ± 0,4
Patient 15	Female	45	Degenerative Disc Disease T12-L1	No	0,40 ± 0,01	0,5981 ± 0,0009
Patient 16	Female	51	Lumbar Canal Stenosis L5-S1	PEEK	0,44 ± 0,03	0,768 ± 0,002
Patient 17	Female	61	Lumbar Canal Stenosis L4-L5	PEEK	1,2 ± 0,2	1,38 ± 0,21
Patient 18	Male	64	Dural Fibrosis L5-S1	No	0,53 ± 0,09	0,88 ± 0,19
Patient 19	Female	52	Lumbar Spondylolisthesis L5-S1	Titanium	0,4 ± 0,1	1,0 ± 0,1
Patient 20	Female	61	Degenerative Disc Disease L4-L5	No	0,9 ± 0,1	0,86 ± 0,02
Patient 21	Male	75	Lumbar Canal Stenosis L5-S1	PEEK	0,908 ± 0,0003	1,4 ± 0,3
Patient 22	Male	46	Degenerative Disc Disease L5-S1	PEEK	0,57 ± 0,03	0,87 ± 0,17
Patient 23	Male	60	Lumbar Canal Stenosis L4-L5	PEEK	0,7 ± 0,1	3,95 ± 0,32
Patient 24	Female	46	Discal Herniation Recurrence L4-L5	Titanium	1,2 ± 0,2	0,773 ± 0,014
Patient 25	Female	33	Degenerative Disc Disease L4-L5	PEEK	1,1 ± 0,4	0,526 ± 0,091
Patient 26	Female	55	Lumbar Spondylolisthesis L4-L5	Titanium	0,48 ± 0,02	2,59 ± 0,01
Patient 27	Male	49	Degenerative Disc Disease L5-S1	No	0,75 ± 0,03	0,64 ± 0,10
Patient 28	Male	46	Lumbar Spondylolisthesis L5-S1	Titanium	0,55 ± 0,07	1,87 ± 0,04
Patient 29	Female	54	Lumbar Canal Stenosis L4-L5	Titanium	0,54 ± 0,01	1,355 ± 0,013
Patient 30	Male	65	Lumbar Canal Stenosis L4-L5	Titanium	0,355 ± 0,002	1,201 ± 0,059
Patient 31	Female	58	Degenerative Disc Disease L5-S1	Titanium	0,54 ± 0,03	1,276 ± 0,047
Patient 32	Female	55	Lumbar Spondylolisthesis L4-L5	PEEK	0,265 ± 0,052	0,704 ± 0,029

**Figure 2:**
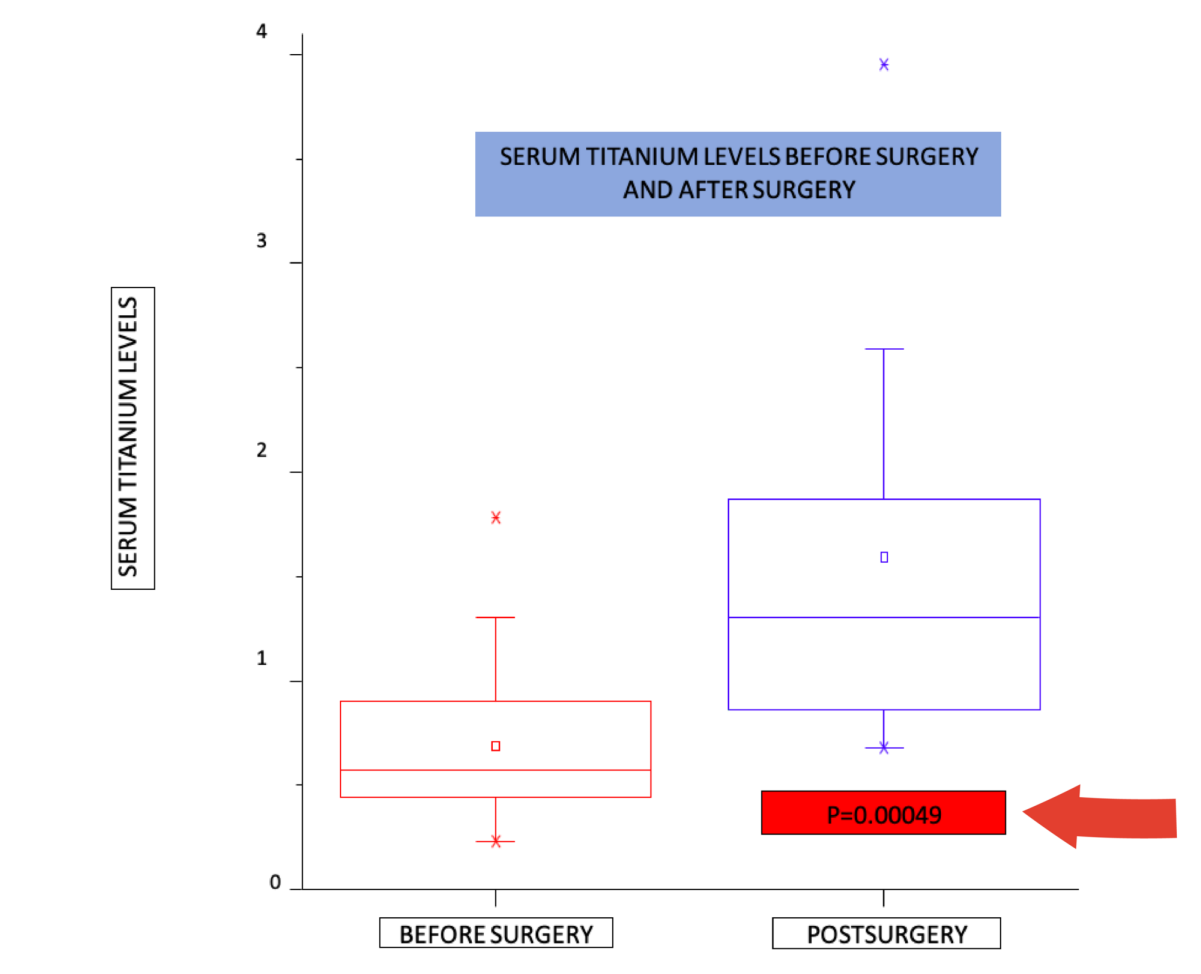
Serum titanium levels before surgery and after surgery

Total titanium determination was conducted in the serum samples of the patients of the different groups undergoing spinal arthrodesis described in the samples section, before and after surgery. Before surgery, the average titanium concentration levels found in the samples are in agreement with previous publications for control samples (0.7449 µgL^-1^, SD=0.562). The average titanium concentration level found after surgery was 2.5406 µgL^-1^ (SD=3,69), a near-3.5-fold increase. The comparative results of the titanium concentration in the serum of 32 patients analyzed before surgery (left bar) and after spinal arthrodesis (right bar) are shown in Figure 2. The time elapsed after surgery was one year. In this case, the average increase in concentration turned out to be 1.7957 µgL^-1 ^(SD=3.5765, Range=-0.57 µgL^-1^; 14.60 µgL^-1^) higher and there is a statistically significant increment (p=0.00049) of the titanium concentration in the serum of the patients after surgery (Figure 2). Serum titanium levels per patient are shown in Table 2.

**Figure 3:**
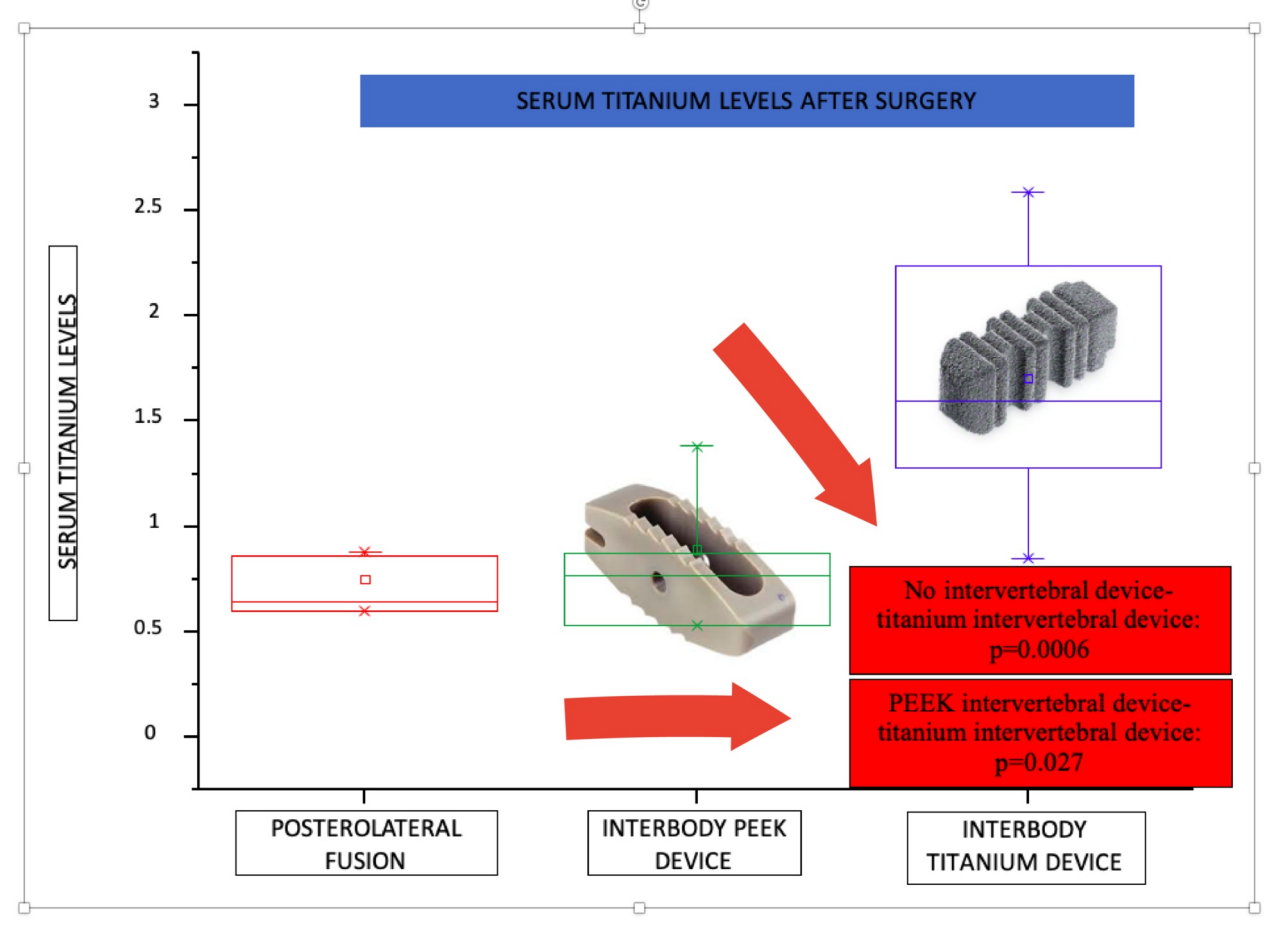
Serum titanium levels after surgery and its distribution by spinal arthrodesis devices PEEK: polyester ether ketone

In addition, it was possible to separate the patients into three groups depending on the type of implant used (posterolateral fusion and 360-degree fusion, out of which some patients carried a PEEK intervertebral device and others a titanium intervertebral device). There are no significant differences between the patients not carrying any intervertebral device and those carrying a PEEK device (p=0.916). However, both of them show statistically different results with respect to patients carrying a titanium-intervertebral device (no intervertebral device-titanium device: p=0.0006 and PEEK intervertebral device-titanium device: p=0.027) (Figure 3).

## Discussion

Immediately after implantation in the human body, all implants start to degrade through two mechanisms: corrosion and wear [17]. Wear is the mechanical/physical degradation of materials (producing particles), whereas corrosion is a chemical (or electrochemical) form of degradation (mainly producing solubilized metal ions). Thus, the use of titanium alloys for biomedical applications has introduced the possibility of generating microscopic titanium particles together with titanium metal ions that are present in the tissues of the surrounding implants (peri-implant environment) [2,7]. The particles are commonly of nanometer size and activate macrophages that increase bone absorption and, eventually, inflammatory reactions leading to the implant failure [4]. The behavior of metal ions released into biological fluids can be diverse, showing combination with biomolecules like proteins (99.8% in serum is bound to transferrin) or with a water molecule or an anion to form an oxide, hydroxide, or inorganic salt [5,15,18].

Metal liberation from metallic implants has been reported in the body fluids of patients carrying total hip or knee replacements, intramedullary nails, and, more recently, in patients undergoing instrumental spinal arthrodesis [2-3,11-12,14-15,19-30].

Blood and serum titanium levels are well-studied and defined in relation to total hip arthroplasty, whether primary, revision, or during follow-up, from one of the first works of Agins in 1988 to the last revision of Swiatkowska [20-21]. Jacobs et al. have found that the normal value for serum titanium levels for individuals without titanium-containing implants is less than 2 µgL^-1^ or parts per billion (ppb) [24]. In a more recent work published by Nuevo-Ordóñez et al. with a detection limit of about 0.05 µgL^-1^, they established the average levels of 40 control individuals at a mean of 0.26 µgL^-1^ [19]. In the same work of Jacobs, et al., the authors have found for a total hip arthroplasty population with well-functioning metal-on-polyethylene implants a serum titanium level approximately of 4 µgL^-1 ^or ppb, and levels greater than 8 µgL^-1 ^or ppb when implants were either subject to loosening or accelerated wear due to abrasion [22].

Metal ion release has been addressed in patients undergoing spinal surgery as correction of the deformity in adolescent idiopathic scoliosis literature or instrumented spinal arthrodesis [2-3,11,25-30]. Localized metal debris has been well-documented in the paraspinal soft tissues surrounding instruments [3,5]. Systemic distribution via blood and lymphatic vessels and distant organ settlement of particulate debris have also been demonstrated [26].

There are few works in the literature on metal ion serum levels after deformity correction surgery. Those on serum titanium levels in patients with early-onset scoliosis or adolescent idiopathic scoliosis reported an increase in mean serum metal ion levels [26-30]. Cundy et al. reported, in two prospective and longitudinal studies, that preoperative and postoperative concentrations of serum titanium and niobium were significantly different (p=0.0001), and median postoperative serum concentrations of titanium and niobium were elevated 2.4- and 5.9-folds above the normal range, respectively. They also observed a significant and rapid rise in serum titanium and niobium levels within the first postoperative week, after which elevated serum levels persisted up to 12 months [26-27]. In another prospective study, Cundy et al. reported that measurable metal ion levels were detected in all local samples obtained from wound irrigation fluid, cell saver blood, and fluid that immersed metal universal reduction screw tabs, and postoperative serum metal ion levels were elevated compared to baseline preoperative levels. Another observation in that study was that, in general, metal ion levels were considerably higher in intraoperative fluid samples compared to those observed in the serum levels. They concluded that their findings of contextually high metal ion concentrations in intraoperative and early postoperative samples provide further empirical support of a "putting-in" phenomenon of metal ion release following instrumented spinal fusion. This complements existing beliefs that metal ion release occurs during an intermediate "wearing-in" phase [28]. Lukina et al., in a retrospective study of implantation of titanium growth guiding sliding instrumentation for scoliosis in children, reported that the content of titanium and vanadium ions in the whole blood of 90% of patients with implanted LSZ-4D^TM^ (CONMET, Moscow, Russia) devices was increased as compared with the control group (2.8 and 4 times, respectively) but did not exceed the values reported previously in the literature for fusion spinal instrumentation. They also observed that the median content of titanium ions in the soft tissues adjacent to the implanted sliding device was measured to be more than 1,500-fold higher as compared with the control group, which is a much higher level than previously reported for spinal instrumentation. No statistically significant difference in metal ion content in the blood was revealed in patients with and without metallosis-associated complications. According to their findings, they recommend that either the use of wear-resistant coatings on titanium alloy sliding devices or the use of a different material for such instrumentation would be beneficial [29]. In a recent prospective study of metal ion release during growth-friendly instrumentation for early-onset scoliosis published by Yilgor et al., they have also reported serum titanium levels higher than controls in all groups of cases (p<0.001) [30].

There are very few studies that analyze serum or blood titanium levels in adults with spine pathologies and spinal fusion, and most of them are retrospective cohorts. Richardson et al. and Kasai et al. reported 3.6-fold and 4-fold increases in the serum titanium levels of adult patients with instrumentation implanted on two or three levels of the lumbar spine [2,11]. Ipach et al. also demonstrate 2 to 3-fold increases of titanium content in adult patients with a median of five fused segments [25].

In the retrospective study measuring postoperative serum titanium levels in 30 patients with titanium alloy spinal instrumentation published by Richardson et al., they observed serum titanium levels were significantly higher in patients with titanium spinal implants (mean=2.6 µgL^-1^) when compared with controls (mean=0.71 µgL^-1^), very similar serum levels to those we found in our study. They reported that subjects who underwent an instrumented arthrodesis of only one spinal segment had decreased serum titanium levels when compared with those who were fused at two or more spinal segments (mean 2.3 versus 3.1 µgL^-1^) and patients with four or fewer pedicle screws also had decreased serum titanium levels when compared with the constructs of six to eight pedicle screws (mean 2.3 versus 3.35 µgL^-1^); however, both of these findings were not statistically significant. Another observation was that patients without cross connectors had a slightly increased serum titanium level when compared with those with connectors (mean 2.7 vs. 2.44 µgL^-1^); however, this finding was also not statistically significant. Also, patients with titanium interbody devices had a statistically significant elevation in serum titanium levels when compared with those without (mean 3.3 versus 1.98 µgL^-1^) [2].

Kasai et al. performed a retrospective study to evaluate serum and hair metal concentrations in patients with titanium alloy spinal implants. Of the 46 patients with titanium alloy spinal implants, 16 patients (34.8%) exhibited abnormal serum metal concentrations and 11 patients (23.9%) exhibited abnormal hair metal concentrations. In the control group, three patients (15%) exhibited only abnormal serum and metal aluminum concentrations at the first examination. Comparison of the implant failure and no implant failure groups showed no significant differences in the incidence of abnormal serum concentrations of titanium, aluminum, or both metals. Therefore, serum metal concentrations did not seem to be a useful indicator of hardware loosening or implant failure [11]. But this study has several drawbacks, such as being retrospective, having a very small number of cases and controls, and only seven of 19 individuals had serum titanium levels because they were beneath the reference value and they did not could report them.

Ipach et al. designed a prospective study to determine changes in whole blood titanium levels after spinal fusion and to analyze the correlation to the number of pedicle screws, cross connectors, and interbody devices implanted. They reported no statistically significant correlation between fused segments (r=-0.188, p=0.503), length of instrumentation (r=-0.329, p=0.231), number of interbody devices (r=-0.202, p=0.291), and increase of titanium levels over the observation period. They observed no statistically significant increase in titanium levels 12 months after surgery (mean difference=-7.2 μgL^-1^, p=0.446) [25]. Those results are totally in contraposition with others in the literature and with ours too since not only do titanium levels not increase, but they decrease after surgery. This result is not well-explained by measuring titanium levels in the blood rather than serum as authors speculate.

In our study, serum titanium levels were found to be higher after surgery in all groups in comparison to levels before surgery. Before surgery, the average titanium concentration levels found in the samples are in agreement with previous publications for control samples (0.7449 µgL^-1^, SD=0.562). The average titanium concentration level found after surgery was 2.5406 µgL^-1^ (SD=3,69), a near-3.5-fold increase. In this study, the average increase in concentration turned out to be 1.7957 µgL^-1 ^(SD=3.5765, Range=-0.57 µgL^-1^; 14.60µg L^-1^) higher, a statistically significant increment (p=0.00049) of the titanium concentration in the serum of the patients after surgery.

Titanium naturally exists in water, soil, and foods. Hence, the normal population is also exposed to such irons, but patients in this study are living in the same geographical regions and similar natural environments [30]. Therefore, it is suggested that they were similarly affected by the non-implant-related metallic environment exposure, although possible individual exposure should also be taken into account. However, when using the same group of individuals as a control and as a case, this possible contamination is minimized. The obtaining and storing of procedures were exact for all patients, and all the samples were sent to the same laboratory. Thus, technical and handling issues equally affected patients in all groups. Therefore, the increase in metal ion levels was attributed to implanted spinal instrumentation.

When we separate the patients into three groups depending on the type of implant used, there are no significant differences between the patients not carrying any intervertebral device and those carrying a PEEK device (p=0.916). Both of them show statistically different results with respect to the patients carrying a titanium-intervertebral device (no intervertebral device-titanium device: p=0.0006, and PEEK intervertebral device-titanium device: p=0.027).

Therefore, the presence of titanium fusion implants increases the circulating titanium levels. These results seem to point out two different facts: the titanium screws and bars produce a significant release of the metal that is further transported into the bloodstream. Additionally, the presence of an intervertebral device does not change the rate of metal release when it is made of PEEK but increases, notably, when this is made of titanium. This could be ascribed to the size of the device that is also in contact with a highly irrigated area. Thus, titanium release might occur due to the electrochemical dissolution process of metallic implants by the blood components since the presence of intervertebral devices that, in principle, reduces the micromotion of the spinal fusion implants (and thus the production of mechanical wear) and increases the level of titanium released when made of this metal. Of course, the presence of a titanium intervertebral device increases the amount of implanted titanium so that a greater amount of titanium surfaces to debris and passes into the bloodstream. Therefore, mechanical fretting does not seem to be the most important cause of the metal release.

However, because this study was cross-sectional, the effect of time cannot directly be judged. The authors point out the need for longitudinal studies to observe and explain time-dependent serum titanium level alterations. Longitudinal studies would also allow the exhibition of ion release differences among posterolateral, circumferential with PEEK, and circumferential with titanium implantations if they exist.

The elevated metal ion levels observed in this study were also comparable to those of the levels in adults with metal-on-metal total hip replacements [20]. Similar to spinal fusion surgery, the initial metal ion release after arthroplasty is followed by a gradual decline until it reaches a plateau. Ion levels tend to rise again in the presence of loosening or mechanical failure. Metal ion exposure can have an impact on liver and kidney functions and trigger immunologic responses even with well-fixed metal-on-metal prostheses. Therefore, in patients undergoing spinal fusion techniques will have exposure to elevated serum titanium levels and there is a potential for long-term deposition of it in organs such as the liver, spleen, lungs, kidneys, and heart. Moreover, the local and systemic long-term clinical effects of increased metal ion levels are yet to be determined. Nonetheless, it is reasonable to think that the suggested various potential deleterious effects would increase as the duration and amount of exposure increases.

There are several drawbacks to this study. The main drawback is that it is a cross-sectional study analyzing a time-dependent event. Intra- and postoperative consecutive blood samples were not obtained, only the sample before surgery and at the one-year follow-up. Sequential data over time for each patient are not present. Additionally, no patients in this series underwent revision procedures so the issues of local staining and corrosion are not known. Furthermore, although a control group was included, epidemiologic data are not available to assess the normal serum titanium levels in a wider population. Yet, this study is the first study related to metal ions that compares different spinal arthrodesis techniques, so it is important that it points toward a subject that requires attention. Further prospective follow-up studies have to be designed to allow deeper and more precise analyses. Time-dependent alterations of serum ion levels and their mechanism, the structural properties of the devices, and exposure to environment exposition warrant investigation.

## Conclusions

Patients undergoing posterior spinal arthrodesis experienced a significant increase in titanium serum concentrations with respect to the concentrations initially detected before surgery. The stabilization of the implant by using an intervertebral disc did not provide any difference in terms of the metal released due to micromotion since the levels of titanium were comparable in the absence of a disc or in the presence of a PEEK disc. However, when the intervertebral disc is made of titanium, it is possible to detect significant increases in the liberated metal.
